# Outcomes of trimethoprim/ sulfamethoxazole treatment for ocular toxoplasmosis in Congolese patients

**DOI:** 10.1186/s12886-023-03183-x

**Published:** 2023-10-31

**Authors:** Nadine Nsiangani Lusambo, Dieudonné Kaimbo Wa Kaimbo, Dieudonné Mumba Ngoyi Mumba, Alejandra de-la-Torre

**Affiliations:** 1 Eye Department, Teaching Hospital, Medical School, University of Kinshasa, Kinshasa, Democratic Republic of Congo; 2 Parasitology Department, Teaching Hospital, Medical school, University of Kinshasa, Kinshasa, Democratic Republic of Congo; 3 Neuroscience Research Group (NEUROS), Neurovitae Center for Neuroscience, Institute of Translational Medicine (IMT), Escuela de Medicina y Ciencias de la Salud, Universidad del Rosario, Bogotá, Colombia

**Keywords:** Ocular toxoplasmosis, Treatment, Outcome, Trimethoprim/Sulfamethoxazole, RD Congo

## Abstract

**Background:**

Ocular toxoplasmosis (OT) is the leading cause of infectious posterior uveitis in several areas worldwide. The combination of Trimethoprim/Sulfamethoxazole (TMP/SMX) has been presented as an attractive alternative to the “classic’ treatment therapy (Pyrimethamine/Sulfadiazine).

**Methods:**

A prospective study was carried out between February 2020 and September 2021 in 2 ophthalmic centers in Kinshasa. This study aimed to describe TMP/SMX treatment outcomes for OT in a cohort of immunocompetent Congolese patients.

**Results:**

54 patients were included, with a mean age at presentation of 37.5 ± 13.6 years old and a Male-Female ratio of 1.45:1. Three patients (5.6%) presented a recurrence during the follow-up period. At the end of the follow-up, improvement in VA and resolution of inflammation concerned 75.9% and 77.5% of patients, respectively. Cataracts (3.7%), macular scars (3.7%), and vitreous opacities (3.7%) were the principal causes of non-improvement in VA. Treatment-related adverse events were present in 10 patients (18.5%); gastrointestinal (14.8%) and dermatological (3.7%) adverse events were the most frequent. Dermatological adverse events led to discontinuation of treatment.

**Conclusion:**

TMP/SMX regimen appears to be a safe and effective treatment for OT in Congolese patients. The low cost and the accessibility of the molecules make this regimen an option for treating OT in resource-limited countries.

## Background

Ocular toxoplasmosis (OT) is the leading cause of infectious posterior uveitis in several geographical areas [1, 2]. It is more common in South America, Central America, The Caribbean, and parts of tropical Africa compared to Europe and Northern America [3]. OT is also a significant cause of visual impairment worldwide, accounting for up to 57% of visual impairment and 24% of blindness in infected persons with ocular involvement [4, 5]. Although OT remains a common and sight-threatening cause of infectious posterior uveitis, treatment remains highly controversial. Up to this day, there is no consensus on treatment effectiveness for the occurrence and recurrence of this disease [6, 7].

In the treatment of OT, ‘classic therapy’ refers to the combination of pyrimethamine, sulfadiazine, and systemic corticosteroids, and this combination has been used for OT treatment since the 1950s [2, 7–9]. However, this classic therapy may not be accessible in some areas and may have significant adverse side effects. Due to this therapy’s intolerance, accessibility, and adverse drug reactions, alternative treatments with better safety profiles have been sought [9]. Recent studies have shown that Trimethoprim/Sulfamethoxazole (TMP/SMX), an alternative to the classic therapy, is an attractive option due to its low cost, wide availability, and tolerability. However, sulfonamide-related reactions may occur [2].

Differences in clinical presentation, parasite strains, and host immune status may explain the individual response to different treatment approaches [7, 10]. Several treatment schemes, based on the inflammatory response, patients’ age, lesion size, and location, are supported by scientific research [10]. The literature in Europe, the United States, South America, and Asia is well known; however, in Africa, there is still a scarcity of knowledge about the response to medications in patients with OT [11]. Therefore, this study aims to describe the outcome of treating ocular toxoplasmosis with a TMP/SMX regimen in a cohort of immunocompetent Congolese patients.

## Methods

A cross-sectional study was conducted in two ophthalmic centers in Kinshasa (Saint Joseph Hospital and Ophthalmic Clinic of Masina), the capital city of the Democratic Republic of Congo (DRC), between February 2020 and September 2021 (20 months).

Patients diagnosed with active OT who agreed to participate in the study and with a follow-up time of at least six weeks were enrolled and examined by the same ophthalmologist. Patients with a history of drug allergy and treated with azithromycin and HIV (Human Immunodeficiency Virus) positive patients were excluded. Detailed ocular and medical records were obtained from each patient. A complete ophthalmic examination was performed, including best-corrected Snellen visual acuity, slit-lamp biomicroscopy, tonometry, and indirect ophthalmoscopy. A follow-up ophthalmic examination was scheduled every two weeks until the resolution of lesion activity.

Antiparasitic treatment with TMP/SMX (160 mg TMP + 800 mg SMX, twice daily) for at least six weeks, depending on the evolution of the lesions, was used in each patient. After 48 h, an oral corticosteroid (prednisolone 1 mg/kg daily) was started. Topical corticosteroids and mydriatic eye drops were used in the case of anterior chamber inflammation. In addition, some patients with significant vitreous inflammation that did not improve with systemic antibiotics combined with systemic corticosteroids received subconjunctival triamcinolone injections.

The diagnosis of OT was based on the presence of characteristic retinochoroiditis lesions in fundus examination, confirmed by serology testing. Primary OT was defined as creamy-white exudative focal retinochoroiditis not associated with retinochoroidal scars in either eye. Recurrent OT was defined as focal active retinochoroiditis associated with retinal scarring in the same or contralateral eye [6, 12].

The evolution of visual acuity (VA) and the resolution of the inflammation were the parameters of the treatment evaluation.

The patient’s visual acuity was classified according to World Health Organization (WHO) criteria [13]. “Baseline visual acuity” was the best-corrected visual acuity (BCVA) measured at the first consultation; “final visual acuity” was the BCVA measured at the end of the follow-up of the affected eye and by a person. Improvement in VA was considered for a gain of at least 1 line on the Snellen scale or for maintaining a VA of 6/6 at the end of follow-up. Worsening of visual acuity was considered for a decrease of at least 1 line on the Snellen scale or for maintaining a visual acuity level of less than 6/12 despite treatment. Improvement in the inflammation was defined as either a two-step decrease in the level of inflammation or a reduction to “inactive,“ and worsening of the inflammation was defined as either a two-step increase in the level of inflammation or an increase to the maximum grade [14]. Ocular hypertension was defined as an IOP above 21 mmHg. Intraocular inflammation was classified according to the Standardization of Uveitis Nomenclature (SUN) workshop and the International Uveitis Study Group criteria [14].

Toxoplasma serology testing (IgG and IgM) was performed on each patient by enzyme-linked fluorescent assay (ELFA, Biomerieux, France).

Results are expressed as mean ± SD [min–max] for continuous variables and frequency (%) for categorical variables. Differences in proportions were analyzed using the chi-squared test, and means were compared using Student’s *t*-test. Statistical Package for Social Sciences (SPSS) software version 21.0 was used for statistical analysis. *P* values less than 0.05 were considered statistically significant.

The Ethics Committee of the Medical School of the University of Kinshasa (ESP/CE/047/2020) approved this study. All patients or their legal tutors for minors signed informed consent to participate in the study.

## Results

### Demographic and clinical characteristics

According to the inclusion criteria, fifty-eight patients were identified, among which four patients with a history of drug allergy and treated with azithromycin were excluded. Men represented 59.3% of the patients, with a M:F ratio of 1.45:1. The mean age at presentation ± SD was 37.5 ± 13.6 years [range: 13–64 years]. The mean duration of the follow-up period was 156.7 days ± 123.9 [range: 45–605 days].

**Fig. 1:**
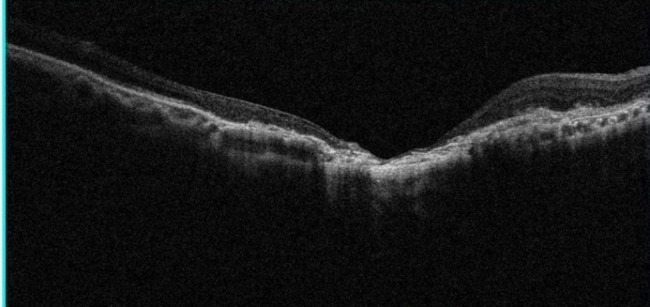
OD optical coherence tomography: large macular retinochoroidal scar in a 49-year-old man with ocular toxoplasmosis

Decrease in visual acuity (98.1%), ocular pain (37%), and floaters (27.8%) were the most common complaints. Ocular involvement was unilateral in 41 patients (75.9%) and bilateral in the others, giving a total of 67 eyes affected. Half of the patients (50%) had primary OT, and the other had a recurrence. Ten patients had macular lesions (18.5%); two of them had bilateral lesions, giving a total of 12 affected eyes. In addition, macular lesions were active in 6 eyes and cicatricial in the others (Fig. 1). The IgG antibodies were positive for all patients (100%); only four of them had positive IgM (7.4%) (Table 1).

**Table 1:** Demographic and clinical characteristics of Congolese patients with OT treated with - TMP/SMX

Characteristics	Frequency (%)
**Sex**	
• Male • Female	32 (59.3) 22 (40. 7)
**Age (years)**	
• < 20 • 20–40 • 41–60 • > 60	5 (9.3) 31 (57.4) 17 (31.5) 1 (1. 8)
**Type of OT** • Primary • Recurrence	27 (50) 27 (50)
**Laterality**	
• Bilateral • Unilateral	13 (24.1) 41 (75.9)
**Localization of lesions**	
• Macula • Macula + periphery • Central outside macula • Central + Periphery • Periphery	8 (14.8) 2 (3.7) 10 (18.5) 4 (7.4) 30 (55.6)
**Ocular complaints**	
• Decrease in VA • Pain • Floaters • Redness • Photophobia • Metamorphopsia • Others	53 (98.1) 20 (37) 15 (27.8) 11 (20.4) 7 (13) 2 (3.7) 5 (9.3)
**Vitreous haze**	
• 0 • 1+ • 2+ • 3+ • 4+	5 (9.3) 18 (33.3) 9 (16.7) 12 (22.2) 10 (18.5)
**Toxoplasma serology**	
• IgG • IgM	54 (100) 4 (7.4)

At the initial consultation, 31 patients (57.4%), with a total of 34 eyes (3 patients with bilateral involvement / 50.7% of eyes) had visual impairment (VA < 6/18), including eight patients (14.8%) with low vision (6/18 > VA ≥ 3/60) and 23 patients (42.6%) with blindness (VA < 3/60). In addition, five patients (9.3%) had no vitreous haze, and 18 (33.3%) had 1 + vitreous haze. (Table 1).

### Treatment and evolution

All patients were treated with TMP/SMX (160 mg TMP + 800 mg SMX, twice daily) combined with oral corticosteroids.

At the end of the follow-up, an improvement in VA (a gain of at least 1 line on the Snellen scale or maintaining of 6/6) was found in 41 patients (75.9%), and 13 (24.1%) patients had worsened in VA. A visual impairment (VA < 6/18) concerned 16 patients (29.6%) and 18 eyes (26.9% of the eyes); two patients (3.7%) had bilateral involvement. Blindness (VA < 3/60) was found in ten patients (18.5%), and one of them had bilateral blindness (1.8%). Blindness was found in 4 of the 12 eyes with macular lesions (33.3%), whereas it only affected 7 (12.7%) of the eyes with lesions outside the macula; the causes of blindness in these 7 eyes are listed in Table 2. In 13 patients (24.1%), VA remained stable or deteriorated during follow-up; among them, three patients had macular lesions. Cataracts (3.7% of patients, 4.5% of eyes), macular scar (3.7% of patients, 2.9% of eyes), and vitreous opacities (3.7% of patients, 2.9% of eyes); were the principal cause of non-improvement in visual acuity in these patients. (Table 2).

**Table 2:** Evolution of Visual acuity during the follow-up

	Patients (N = 54)	Affected Eyes (N = 67)
**Initial BCVA**		
• Normal • Low vision • Blindness	23 (42.6%) 8 (14.8%) 23 (42.6%)	33 (49.3%) 8 (11.9%) 26 (38.8%)
**Final BCVA**		
• Normal • Low vision • Blindness	36 (66. 7%) 8 (14. 8%) 10 (18. 5%)	49 (73. 1%) 8 (11. 9%) 10 (15%)
**Evolution of VA**		
• Improvement • No improvement	41 (75.9%) 13 (24.1%)	53 (79.1%) 23 (20.9%)
**Causes of no improvement of VA**		
• Cataract • Macular scar • Vitreous opacities • Macular edema • Optic atrophy • Epiretinal membrane	2 (3.7%) 2 (3.7%) 2 (3.7%) 2 (3.7%) 1 (1.8%) 1 (1.8%)	3 (4.5%) 2 (2.9%) 2 (2.9%) 1 (1.5%) 1 (1.5%) 1 (1.5%)

Of the 49 patients with vitreous inflammation, 38 (77.5%) showed a resolution of inflammation at the end of follow-up. The mean time to resolve vitreous inflammation was 79.1 ± 48.38 days (limits: 20–217 days). The time of resolution of vitreous inflammation was statistically longer for patients with a haze of at least 2 + compared to those with a haze of 1+ (96.2 ± 51.6 days VS 55.4 ± 31.7 days, *P* = 0.005).

Significant vitreous inflammation, not resolving despite the systemic antibiotic combined with systemic corticosteroids, required subconjunctival triamcinolone injections in 30 patients (55.6%). Patients who received subconjunctival triamcinolone were treated with TMP/SMX prophylaxis for at least six months after the injection.

Although the use of subconjunctival triamcinolone injections remains questionable, we found that the frequency of this therapy in our practice increased with the severity of vitreous haze at the initial consultation (*P* < 0.001). A favorable change in visual acuity was more frequent among patients who had not received triamcinolone; however, the difference was not statistically significant (79.2% VS 70%, *P* = 0.44). Cataracts were significantly more frequent among subjects who received triamcinolone than others (33.3% VS 8.3%, *P* = 0.02).

In 18 patients (33.3%), eye drops for ocular hypertension were used during the follow-up. Among these 18 patients, 10 (10/54 patients = 18.5%) presented ocular hypertension at the initial consultation, which improved with treatment except for one patient (1.8%). For the eight other patients, ocular hypertension appeared during follow-up after triamcinolone injection; it was transient in 7 (12.9%) of them and persisted in 1 patient.

Three out of 54 patients (5.6%) presented a recurrence during the follow-up period. The delays of these recurrences were respectively four, six, and eight months.

### Treatment-related adverse events

Ten patients developed treatment-related adverse events (18.5%). Gastrointestinal adverse events were the most common and concerned eight patients (14.8%). Dermatologic adverse events affected two patients (3.7%) (Table 3). Among the patients with gastrointestinal adverse events, prescribing antacids was necessary only for two. In none of these patients antiparasitic treatment was stopped. The presence of dermatologic adverse events led to treatment discontinuation in the two patients (3.7%). Dermatologic manifestations regressed spontaneously on discontinuation of antiparasitic treatment.

**Table 3:** Treatment-related adverse events

	N (%)
**Gastrointestinal adverse events** Epigastralgia Constipation Nausea	8 (14.8%) 6 (11.1%) 1 (1.8%) 1 (1.8%)
**Dermatologic adverse events** Pruritus Skin rash	2 (3.7%) 1 (1 8%) 1 (1.8%)
**Total**	10 (18.5%)

### Cost of treatment

The cost for a week of treatment with the TMP/SMX combination was USD 15.3, and the total cost for a 6-week treatment was USD 91.85. On average, one week of treatment with the classic regimen (pyrimethamine, sulfadiazine, and folinic acid) costs USD 89.7, and for six weeks, the total cost is USD 358.9.

## Discussion

In the DRC, OT is the leading cause of infectious uveitis [15]. In addition, it has a significant visual impact because retinochoroidal lesions affect the posterior pole in 74% of patients [16]. Therefore, adequate management of these patients is essential to reduce the risk of visual impairment.

Available treatments for OT include a combination of pyrimethamine and sulfadiazine plus corticosteroids as a classic and standard treatment [2, 7, 8, 17], oral clindamycin (alone or in combination with the classic therapy), TMP/SMX, azithromycin, ubiquinone analogs (atovaquone), and intravitreal injection of clindamycin [2, 18]. However, there is no agreement regarding the best drug combination, and few patient-based studies have compared the efficacy of different drugs [19].

The current therapies for OT have not shown a complete cure since the principal achievement of the treatment is to limit parasite multiplication, which relates to ameliorating retinal and optic nerve tissue damage [20]. Visual impairment due to recurrences, vitritis, macular compromise, or complications such as retinal traction and retinal detachment, among others, could be expected in infection by virulent strains [20, 21]. Furthermore, tachyzoites released from reactivated tissue cysts may spread to other retina sites with a recurrence risk. For these reasons, treating any active lesion should be considered [8, 18, 20, 22].

In the DRC, as in most countries, classic therapy has been the option for treating OT for years. However, several factors, such as the high cost, low availability on the market, the large number of tablets, and the potential risk for serious side effects, may lead to the discontinuation of this treatment. Therefore, TMP/SMX treatment has become an attractive option due to its low cost, wide availability, and tolerability [2, 17, 23]. Even though adverse effects like fever, gastrointestinal upset, weight loss, Stevens-Johnson syndrome, toxic epidermal necrolysis, pancreatitis, serum sickness, hyperkalemia, and thrombocytopenia have also been reported [2]. Nevertheless, over time, this combination seems to be increasingly used in the treatment of OT. In 1991, only one Uveitis specialist in the USA reported TMP/SMX as his preferred medical treatment for patients with OT; by 2001, this number had risen to 23% [8]. In Brazil, the most commonly prescribed treatment for OT was TMP/SMX, used by 57% of uveitis specialists [19]. Therefore, we conducted this study to assess the evolution under treatment of Congolese patients with OT.

Although the criteria for starting treatment for OT cases vary according to specialists and countries, all our patients received antiparasitic treatment. Several surveys of uveitis specialists indicate that even experts differ in their therapeutic approaches [7]. Whereas some ophthalmologists will only care for sight-threatening lesions, others will treat all lesions independent of their location [7, 17, 22]. Despite limited evidence of treatment effects, uveitis specialists are more likely to treat patients with OT than in the past [7, 8]. In surveys on members of the American Uveitis Society in 1991 and 2001, 6% and 15%, respectively, of the respondents treated all patients with ocular toxoplasmosis, regardless of the ocular findings. In Germany, India, and Brazil, specialists treated all patients with active disease in 45%, 62%, and 68%, respectively [19].

In immunocompetent patients, the host immune response contributes substantially to the intraocular inflammation that follows tachyzoite replication within the retina; this inflammation is also responsible for ocular damage. For this reason, systemic corticosteroids are probably beneficial to patients with OT, and they are frequently added to the anti-microbial cocktail in immunocompetent patients with toxoplasmic retinochoroiditis, although the doses employed, and timing of administration vary widely between uveitis specialists [8, 17]. The authors of a nonrandomized retrospective evaluation on the effectiveness of different treatments for OT found significant improvement in VA when antibiotics were associated with steroids rather than administered alone [17]. In Brazil, systemic steroids were associated with anti-toxoplasmic therapy in most cases by 51% of the specialists [19]. In a documentary study conducted at the University of Sao Paulo, oral corticosteroids were used in conjunction with antitoxoplasmic medication in 76.3% of patients [20]. In our cohort, we added oral corticosteroid therapy to antiparasitic treatment in all patients.

Additionally, to reduce vitreous inflammation, periocular injection of triamcinolone was indicated in 55.6% of our patients. In Brazil, local treatment (periocular or intraocular) with corticosteroids is indicated for selected patients adjunctively to anti-toxoplasmic therapy by 49% of specialists. In contrast, in the United States, periocular corticosteroid injections are an unpopular approach [8, 19]. The administration of local corticosteroids has been associated with disastrous outcomes if administered without concomitant antiparasitic therapy [8], and the administration of steroids alone can result in fulminant toxoplasmosis responsible for legal blindness in most cases [8, 17, 22]. In our series, we used triamcinolone, a long-acting corticosteroid [24]. Some authors have reported cases of fulminant OT with intravitreal triamcinolone if antiparasitic treatment is not associated [25, 26]. In our series, the patients who received triamcinolone were all under antiparasitic treatment, and we had no case of fulminant OT. Two authors have also reported using intravitreal triamcinolone in immunocompetent and immunocompromised patients with OT and under parasitic treatment, with the resolution of inflammation and improvement in visual acuity [27, 28].

Ocular hypertension was noted in 33.3% of our patients; it was present at the initial consultation in 18.5%. Transient hypertension appeared during follow-up in 12.5% of patients, possibly related to corticosteroid therapy. At the end of the follow-up, only two patients (3.7%) had persistent high intraocular pressure (IOP). Likewise, the literature reported an elevated IOP in 30% of patients with OT at initial examination [7]. In their series, Westfall et al. noted an elevation of IOP at the initial consultation in 38% of patients. This hypertension persisted after the resolution of the episode of OT in 3.3% of them [29].

At the end of the follow-up, an improvement in VA was noted in 75.9% of our patients, and the resolution of inflammation in 77.5%. At the initial consultation, 57.4% of our patients had visual impairment. At the end of the follow-up time, this number had decreased to 29.6%, with 11% cases of low vision and 18.5% cases of blindness. Blindness was more frequent among patients with macular lesions (33.3%) than those without macular involvement (12.7%). Although, in our series, improvement in visual acuity was more significant among patients who had not received triamcinolone, the difference was not statistically significant (*P* = 0.44). The frequency of the occurrence of cataracts was significantly higher (*P* = 0.02) among patients who received triamcinolone, which could explain the less favorable evolution of visual acuity in this group of patients. Cataract is described as one of the most common side effects of triamcinolone, which may affect 14% of treated eyes [24, 30].

Despite treatment, no improvement in VA was noted in 13 of our patients (24.1%). Cataracts (3.7%), macular scars (3.7%), and vitreous opacities (3.7%) were the most common cause of non-improvement of VA among our patients.

In a retrospective study carried out in Brazil by Casoy et al., irrespective of the treatment regimen prescribed, there was a complete resolution or an improvement in the active ocular lesion in 63.9% of patients, and improvements in vision were observed in 56.3% of the overall patient sample [20]. Moreover, in a prospective randomized, single-blind clinical trial carried out to compare the efficacy of the classic therapy of OT versus a regimen consisting of TMP/SMX plus prednisolone, active toxoplasmosis retinochoroiditis resolved in all patients over six weeks of treatment, with no significant difference in mean reduction of retinochoroidal lesion size between the two treatment groups. Similarly, the two groups found no significant difference in VA after treatment. There was also an insignificant difference in the reduction of vitreous inflammation between groups [31]. Also, in the meta-analysis conducted by Zhang et al., they suggested that when patients are intolerant to pyrimethamine + sulfadiazine, TMP/SMX may be considered as an alternative drug in improving VA, controlling vitreous inflammation, reducing recurrence, and improving drug compliance [9].

In our series, treatment-related adverse events were noted in 18.5% of patients. Gastrointestinal adverse events were the most common (14.8%), while dermatologic adverse events concerned two patients (3.7%). All these adverse events were non-serious and resolved. Using real-world data reported to the FDA adverse event reporting system (FAERS), adverse outcomes associated with the treatment of *Toxoplasma gondii* infections in patients with various health backgrounds were analyzed, and most of them were caused by pyrimethamine (27% of adverse effects), followed by sulfonamides containing drugs (20% of adverse effects). Additionally, most serious reports were associated with pyrimethamine (26% of serious adverse events). Among the reported cases, those occurring in patients with OT represented only 2% [32].

In a systematic review of 11 studies about the adverse event profile of pyrimethamine-based therapy for OT, treatment-related adverse events (AE) were reported from 2.3 to 100%. Gastrointestinal-related AE were reported in four studies and included diarrhea, gastrointestinal distress, nausea, vomiting, and loss of appetite. Eight studies reported dermatologic AE, including skin rash, pruritus, and Steven–Johnson syndrome. Skin rash was the most common dermatologic AE (2.8–11%) [33]. Soheilian et al. found no difference in AE profile between patients on classic therapy and those on the TMP/SMX regimen. In their series, the only drug reaction in both groups was the development of a skin rash [31]. Zhang et al. found that TMP/SMX was the most effective intervention with less AE than other treatments [9]. Compared to the classic therapy, TMP/SMX seems to be a good alternative treatment of OT in immunocompetent and immunosuppressed patients, particularly since this is associated with an acceptable side-effect profile and prevention of recurrences as prophylactic treatment [1, 34–36].

In our series, discontinuation of antiparasitic treatment was indicated in two patients (3.7%) with dermatologic adverse events. In Ben-Harari et al. systematic review of the adverse event profile of pyrimethamine-based therapy, it was noted that discontinuation or change in treatment due to adverse events was reported in a range of 0 to 26% in different studies [33].

The cost for a week of treatment with the TMP/SMX combination was USD 15.3, and the total cost for a 6-week treatment was USD 91.85. On average, one week of treatment with the classic regimen (pyrimethamine, sulfadiazine, and folinic acid) costs USD 89.7. For six weeks, the total cost is USD 358.9, without considering the cost of regular hematological controls to follow pyrimethamine’s possible hematological side effects. In the DRC, patients do not have health insurance and must pay on their own for their health care; hence, the cost of the treatment is an essential element to consider in choosing the therapeutic regimen. The availability of antiparasitic drugs is another factor to consider; in our settings, the molecules of the classic therapy are not readily available. In Colombia, a study investigating the cost-effectiveness of four first-line treatment regimens found that TMP/SMX had the best performance. This antibiotic treatment has been established as an economical alternative, and its effectiveness is similar to the classic therapy for active toxoplasmic retinochoroiditis [23]. Nevertheless, there is no consensus about the best treatment regimens in OT. The selection of therapy regimens must be made individually, considering the safety of each therapeutic regimen, medical history of sulfa allergy, and the availability of medications offered within each nation’s health system [18].

Although our study is the first to describe the treatment of OT in patients from sub-Saharan Africa, we recognize some limitations. For example, the retrospective and descriptive approaches limit the comparison between the treatment regimens; comparative studies could provide more significant insights. In addition, the small sample limits the generalization of our results.

## Conclusions

The treatment of OT in Congolese patients with the TMP/SMX plus corticosteroids regimen appears to be effective, improving visual acuity and healing the retinochoroiditis lesions in most patients. Additionally, treatment-related side effects concern 1/5 of patients, most of which appear to be minor, leading to treatment discontinuation in a minority of cases. The affordable cost, the accessibility of the molecules, and the simplicity of posology can motivate using this alternative regimen for OT in our setting. To evaluate the effectiveness and safety, randomized controlled trials of non-inferiority between therapeutic regimens available for treating OT must be done to improve disease management.

## Data Availability

The datasets used and/or analysed during the current study are available from the corresponding author on reasonable request.

## List of abbreviations

OT: Ocular toxoplasmosis
TMP/SMX: Trimethoprim/Sulfamethoxazole
DRC: Democratic Republic of Congo
HIV: Human Immunodeficiency Virus
VA: Visual Acuity
WHO: World Health Organization
BCVA: Best-corrected visual acuity
ELFA: Enzyme-linked fluorescent assay
SPSS: Statistical Package for Social Sciences
FAERS: FDA adverse event reporting system
AE: Adverse events
